# A Hyperbranched Rolling Circle Amplification Assay Using Aptamer–Hemin/G-Quadruplex Recognition for the Detection of Creatine Kinase-MB

**DOI:** 10.1021/acsmeasuresciau.6c00016

**Published:** 2026-07-01

**Authors:** Ensiyeh Mirsadoughi, Sayantan Tripathy, Fatemeh Zare, Gerard L. Cote, Samuel Mabbott

**Affiliations:** † Department of Biomedical Engineering, 14736 Texas A&M University, 101 Bizzell Street, College Station, Texas 77843, United States; ‡ Center for Remote Health Technologies & Systems, Texas A&M Engineering Experiment Station, 600 Discovery Drive, College Station, Texas 77840-3006, United States; § Department of Electrical and Computer Engineering, Texas A&M University, College Station, Texas 77840, United States

**Keywords:** myocardial infarction, CK-MB, aptamer, HRCA, colorimetric

## Abstract

Creatine kinase-myocardial band (CK-MB) remains an important biomarker for the early diagnosis and clinical management of myocardial infarction. Here, we report a sensitive colorimetric assay for CK-MB based on a conformation-locked aptamer guanine-quadruplex (G-quadruplex) system integrated with magnetic nanoparticle capture and hyperbranched rolling circle amplification (HRCA). Target binding induces conformational unlocking of the aptamer, initiating HRCA-mediated signal amplification and generating a concentration-dependent colorimetric response in the presence of a chromogenic substrate. A compact 3D-printed imaging module, combined with a Python-based processing pipeline, was used to extract grayscale intensity values from assay images for quantitative analysis. Using only 25 μL of sample, the assay achieved limits of detection (LOD) of 0.268 ng/mL in aqueous samples and 0.328 ng/mL in blood-spiked samples, and limits of quantification (LOQ) of 0.89 ng/mL and 1.09 ng/mL, respectively, with a linear dynamic range of 0.5–100 ng/mL. Grayscale intensity was inversely proportional to CK-MB concentration, enabling quantitative detection across clinically relevant levels. The HRCA amplification process and target-induced activation were further validated by TapeStation gel electrophoresis, confirming the formation of amplified DNA nanostructures. This platform provides a low-volume, amplification-based strategy for CK-MB detection that is compatible with simple optical readout and has strong potential for point-of-care applications.

## Graphical abstract

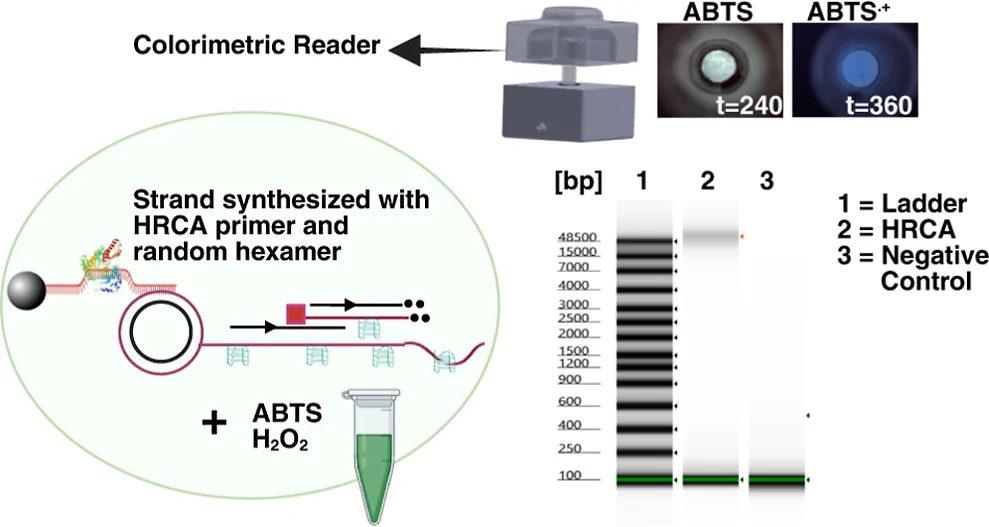

## 1 Introduction

Acute myocardial infarction (AMI) or heart attack is a cardiac event that remains a major cause of emergency visits and deaths.^1^ According to the 2025 update of the American Heart Association (AHA), approximately 805,000 heart attacks occur annually in the U.S., including about 605,000 first-time and 200,000 recurrent events.^2^ The positive impact of a swift and efficient response to AMI has been underscored by decades of research.^3^ AMI is initially evaluated using electrocardiography (ECG), which is highly effective for identifying ST-segment elevation myocardial infarction (STEMI) through characteristic ST-segment elevations that reflect acute disruption of coronary blood flow and the resulting myocardial injury.^4^ Another form of AMI is non-ST-segment elevation myocardial infarction (NSTEMI), which arises from partial or transient coronary occlusion and results in myocardial injury. In contrast to STEMI, NSTEMI often lacks diagnostic ST-segment changes on ECG, leading to substantially reduced sensitivity for ECG-based detection. Consequently, biochemical cardiac biomarkers are routinely used in combination with ECG to confirm the presence of myocardial injury and establish the diagnosis of AMI.

Diagnosis of AMI relies on the measurement of cardiac biomarkers released into the circulation following myocardial injury. Cardiac troponins (cTnI and cTnT) are the current gold standards because of their high sensitivity and cardiac specificity;^5,6^ however, their concentrations typically peak 16–30 h after symptom onset and may remain elevated for up to 2 weeks, which can limit their utility for early detection and for distinguishing recent from prior injury.^7^ Early release biomarkers such as myoglobin and heart-type fatty acid-binding protein (H-FABP) can be detected within the first few hours of symptom onset but lack diagnostic specificity because they are also present in skeletal muscle.^8^ Creatine kinase-myocardial band (CK-MB), a cardiac-enriched isoenzyme of creatine kinase, provides a clinically valuable compromise between early release and tissue specificity. CK-MB typically becomes detectable within 3–6 h after symptom onset, peaks within 12–24 h, and returns to baseline within 2–3 days, making it well-suited for identifying acute myocardial injury and for differentiating recent infarction from older events.^9^ In human serum, CK-MB concentrations below approximately 7 ng/mL are considered normal, while elevated levels are associated with myocardial injury.^10^

Given the important role of CK-MB in AMI diagnosis, several laboratory-based assays have been developed, each with its advantages and disadvantages (See Table 2). The Roche Cobas e411, offers precise CK-MB quantification with high sensitivity.^11^ However, conventional immunoassays typically require processed serum or plasma and trained personnel, resulting in turnaround times of 1–3 h from sample collection to reported results. Furthermore, its reliance on temperature-sensitive reagents, stable infrastructure, and large instrument footprint (>100 kg) confines its use to centralized laboratories.^12^ These constraints limit the suitability of conventional immunoassays for emergency settings, where clinical triage decisions often must be made within 30–60 min^13^ These limitations have motivated the development of point-of-care (POC) strategies for CK-MB detection.^14^

Diagnostic assays, including POC assays, rely fundamentally on selective biorecognition elements to distinguish the target analyte from the surrounding biological matrix.^15^ Antibodies are the most widely used recognition molecules for this purpose because of their high affinity and specificity for their cognate targets. However, antibodies can exhibit limited stability under extremes of temperature and pH, and their random orientation upon surface immobilization, particularly on paper-based substrates, can reduce effective target accessibility. Accordingly, aptamer-based recognition elements have been increasingly adopted in biosensor platforms because of their chemical stability, synthetic reproducibility, and ease of modification.^16^ Aptamers are short, single-stranded DNA or RNA oligonucleotides selected by the systematic evolution of ligands by exponential enrichment (SELEX) process to bind specific targets with high affinity and specificity.^17^ To improve aptamer stability and signal control, conformation-locked aptamer designs based on molecular beacon architectures have been developed.^18^ In this design, the aptamer is initially hybridized with a short complementary blocking strand, which stabilizes it in an inactive conformation and suppresses nonspecific target interactions.^19^ Upon target binding, the aptamer undergoes a conformational rearrangement that displaces the blocking strand, thereby activating target recognition.^20–22^ Although this mechanism enhances specificity, the resulting signal change is often insufficient for the sensitive detection of low-abundance biomarkers such as CK-MB.^23^ To address this limitation, aptamer-based nucleic acid amplification strategies have been employed to enhance detection sensitivity.^23^ Among available amplification approaches, isothermal nucleic acid amplification techniques (iNAATs) have emerged as powerful tools for biosensing, offering high sensitivity while avoiding the need for thermal cycling.^24^ Rolling circle amplification (RCA) is a prominent iNAAT that is particularly attractive because of its isothermal operation, high amplification efficiency, and compatibility with POC formats.^25^

Here, we report a conformation-locked aptamer platform for the selective and sensitive detection of CK-MB. The CK-MB-binding aptamer is immobilized on magnetic nanoparticles (MNPs), enabling target capture and isolation from whole blood.^26^ Figure 1A illustrates the working principle of our conformation-locked aptamer platform for CK-MB detection. We immobilized a CK-MB-specific aptamer onto MNPs to enable efficient target capture and magnetic separation. To minimize background amplification, we deliberately designed the aptamer such that the internal splint sequence remains concealed within its folded structure in the absence of CK-MB, thereby preventing nonspecific hybridization with the circular padlock probe. Upon binding to CK-MB, the aptamer undergoes a conformational rearrangement that exposes the splint sequence, allowing hybridization with the circular padlock probe and initiation of the first amplification stage by EquiPhi29 DNA polymerase in the presence of dNTPs and random hexamer primers. This reaction generates long single-stranded DNA products containing multiple guanine-rich repeat sequences capable of folding into G-quadruplex structures. To further enhance signal generation, we deliberately designed a hyperbranched rolling circle amplification (HRCA) primer that hybridizes to the primary amplification products and initiates a second amplification stage, producing additional DNA strands and increasing the number of guanine-rich motifs. Furthermore, the remaining unreacted random hexamer primers can anneal to newly generated templates and initiate additional extension events, resulting in further amplification and increased production of guanine-rich sequences. In the presence of hemin, these G-quadruplex structures assemble into peroxidase-mimicking hemin G-quadruplexes that catalyze the oxidation of chromogenic substrate 2,2′-azino-*bis* (3-ethylbenzothiazoline-6-sulfonic acid) (ABTS) by hydrogen peroxide (H_2_O_2_), generating a colorimetric signal proportional to the concentration of CK-MB. Signal intensities were quantified using a portable imaging module that extracts red, green, and blue (RGB) values. Using aqueous samples, the assay achieved a limit of detection (LOD) of 0.268 ng/mL and a limit of quantification (LOQ) of 0.89 ng/mL. In blood-spiked samples containing CK-MB in the range of 0.5–100 ng/mL, the assay achieved an LOD of 0.328 ng/mL and an LOQ of 1.09 ng/mL, demonstrating sensitivity across clinically relevant concentrations.

**Figure 1:**
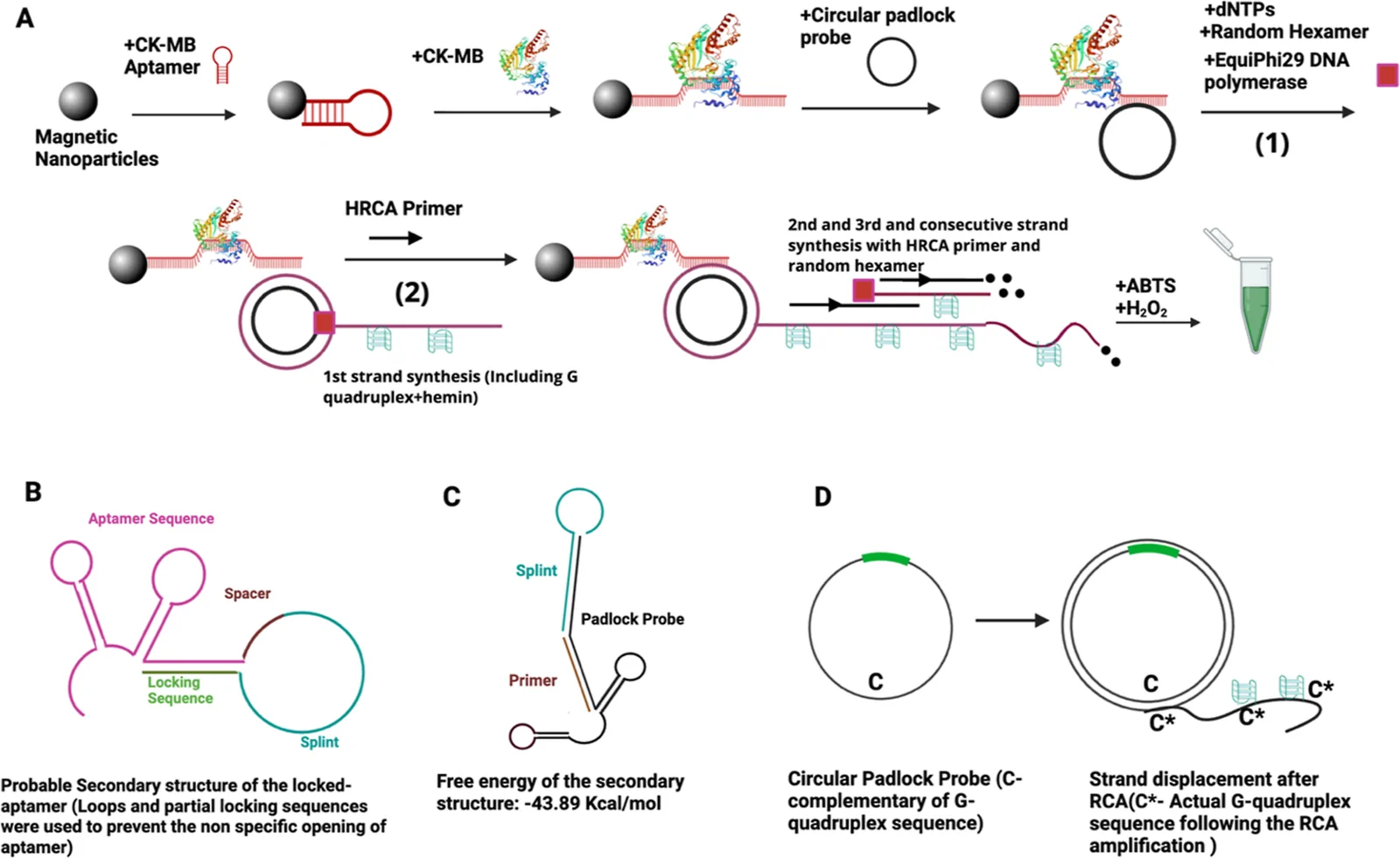
HRCA-based assay design for CK-MB detection. (A) Schematic of the assay workflow. A CK-MB-binding aptamer immobilized on streptavidin-coated MNPs undergoes a target-induced conformational change that exposes an internal splint sequence, enabling hybridization and circularization of a padlock probe. In the first step (1), dNTPs, random hexamer primer, and EquiPhi29 DNA polymerase initiate strand synthesis, generating the first DNA strand and forming G-quadruplex structures in the presence of hemin. In the second step (2), both the HRCA primer and random hexamer primer initiate HRCA, producing multiple DNA strands containing repeated G-quadruplex motifs. In the presence of ABTS and H_2_O_2_, these G-quadruplex structures catalyze a colorimetric reaction, resulting in a green signal. (B) Secondary structure of the engineered conformation-locked aptamer, in which a locking sequence hybridizes to an internal splint sequence to maintain a closed, inactive state. A spacer region reduces steric hindrance and enables target-induced unlocking. (C) Predicted secondary structure of the aptamer-padlock probe complex generated by NUPACK (minimum free energy = −43.89 kcal mol^–1^). (D) Schematic of strand displacement during RCA and formation of G-quadruplex motifs.

## 2 Experimental Section

### 2.1 Materials and Methods

T4 DNA ligase buffer (10×, 1 mL; cat. no. B0202A, lot no. 10270116), T4 DNA ligase (400,000 U/mL, 0.25 mL; cat. no. M0202; lot no. 10269500), deoxynucleotide triphosphates (dNTPs, 10 mM, 0.8 mL; cat. no. N0447, lot no. 10262265), Exonuclease III buffer (cat. no. B7001S), and Exonuclease III (100,000 U/mL, 0.25 mL; cat. no. M0206S/L; lot no. 10266151) were purchased from New England Biolabs (USA). Random hexamer primer (120 μL, 0.2 μg/μL; cat. no. N8080127), adenosine triphosphate (ATP, 100 mM, 25 μmol; cat. no. R0441; lot no. 2998633), dithiothreitol (DTT, 0.1 M, 0.25 mL; cat. no. 707265 ML, lot no. 10266151), 10× EquiPhi29 buffer (cat. no. B39; lot no. 2992436), EquiPhi29 DNA polymerase (10 U/μL, 10 μL; cat. no. A39390; lot no. 3029198), and Dynabeads MyOne Streptavidin C magnetic nanoparticles (MNPs, 10 mg/mL, 1 mL; cat. no. 65001; lot no. 01239648), and 3B Scientific Artificial Blood (Catalog No. S00283) were obtained from Invitrogen (Thermo Fisher Scientific, USA). Nuclease-free water (NFW; cat. no. 11-05-01-04) was purchased from Integrated DNA Technologies (IDT, USA). Active native human CK-MB (cat. no. CKMB-256H) was obtained from Creative BioMart (USA). Cardiac troponin I (cTnI; cat. no. Z03320), cardiac troponin T (cTnT; cat. no. V00402), and myoglobin (cat. no. V00502-1) were purchased from GenScript (USA). Toluene (cat. no. 244511), (3-aminopropyl) triethoxysilane (APTES; cat. no. 440140), *Tris* hydrochloride (*Tris*–HCl; cat. no. T3253), sodium chloride (NaCl; cat. no. S9888-25G), 2,2′-azino-*bis* (3-ethylbenzothiazoline-6-sulfonic acid) (ABTS; cat. no. A1888-1G), hydrogen peroxide (H_2_O_2_, 30% w/w; cat. no. H1009), bovine serum albumin (BSA; cat. no. A2153), sodium phosphate dibasic (Na_2_HPO_4_, anhydrous; cat. no. 342483), potassium phosphate monobasic (KH_2_PO_4_; cat. no. P5655), sodium acetate (cat. no. 241245), potassium chloride (KCl; cat. no. P3911), hydrochloric acid (HCl; cat. no. 320331), sodium hydroxide (NaOH; cat. no. S5881), and ethylenediaminetetraacetic acid (EDTA; cat. no. E9884) were purchased from Sigma-Aldrich (St. Louis, MO, USA). Nanosep centrifugal filters (Omega modified PES, MWCO 3 kDa) were obtained from Cytiva (Marlborough, MA, USA). The NanoDrop One microvolume spectrophotometer and the Agilent 4200 TapeStation system were obtained from Thermo Fisher Scientific and Agilent Technologies (USA), respectively. Ultra-Violet (UV–vis) spectroscopy was performed using an Infinite M Nano + plate reader (Tecan, Switzerland).

### 2.2 SPSC Buffer Preparation

A 1× sodium phosphate-sodium chloride (SPSC) buffer was prepared by dissolving 8.0 g NaCl, 1.44 g Na_2_HPO_4_, 0.24 g KH_2_PO_4_, and 0.2 g KCl in 800 mL of deionized water. The pH of the solution was adjusted to 7.4 using HCl or NaOH. The final volume was then adjusted to 1 L with deionized water. The buffer was sterilized by autoclaving at 121 °C for 20 min and stored at room temperature until use.

### 2.3 Ligation Assay

Padlock probe ligation was performed using a T4 DNA ligase-based protocol to facilitate the circularization of the linear padlock probe in the presence of its complementary splint. For this purpose, 16 μL of padlock probe (10 mM) was mixed with 16 μL of splint. Subsequently, 5 μL of 1× ligase buffer was added. To enable ligation and formation of a phosphodiester bond, 1 μL of ATP, 0.5 μL of DTT, 4 μL of T4 DNA Ligase, and 7.5 μL of NFW were added to the mixture. The reaction was then incubated at 25 °C for 120 min in a PCR thermocycler.

### 2.4 Exonuclease Treatment

To remove unligated DNA, exonuclease digestion was carried out in a 50 μL reaction containing 15 μL of ligated sample (10 μM) obtained from the ligation assay, 5 μL of 10× Exonuclease buffer, 3 μL of Exonuclease III, 1.2 μL of DTT, 25.8 μL of NFW. The mixture was heated at 37 °C for 30 min, followed by rapid cooling on ice for 5 min.

### 2.5 HRCA Mastermix Preparation

To initiate the HRCA reaction, a 20× amplification Mastermix was prepared by combining 60 μL of primer, 200 μL of EquiPhi29 buffer, 10 μL of EquiPhi29 DNA polymerase, 200 μL of dNTP mix, and 1480 μL of NFW, resulting in a total volume of 1950 μL. This Mastermix was designed to support efficient isothermal amplification via strand displacement activity of EquiPhi29 DNA polymerase. Once prepared, the Mastermix was aliquoted into reaction tubes containing the ligated padlock probes, and the amplification was performed under optimal conditions for EquiPhi29 DNA polymerase to facilitate the generation of long, branched DNA products. The HRCA products were subsequently subjected to downstream detection steps.

### 2.6 Preparation of CK-MB-Aptamer Complexes

To prepare MNP-aptamer conjugates, 60 μL of MNPs were suspended in 200 μL of SPSC buffer and incubated with 30 μL of aptamer (prepared by diluting 10 μL of splint in 90 μL of water) for 30 min. After incubation, the supernatant was removed, and 200 μL of 1% BSA was added to block nonspecific binding sites. After 15 min, the supernatant was removed again and replaced with 300 μL of SPSC buffer. For detection, 20 μL of MNP-aptamer conjugates were incubated with CK-MB to obtain final concentrations of 100, 75, 50, 25, 10, 5, and 1 ng/mL. CK-MB working solutions were prepared by serial dilution of a 10 μg/mL stock solution to 1 μg/mL, 100 ng/mL, and 2 ng/mL. For each assay, 1 μL of CK-MB solution was added to the MNP-aptamer conjugates. Subsequently, 2 μL of exonuclease-treated padlock probe was added. After 15 min of incubation, the mixture was washed once with 15 μL NFW using magnetic separation. Then, 9 μL of NFW, 2.5 μL of hemin, 1 μL of random hexamer primer, and 12.5 μL of 1× Mastermix were added, and the mixture was subjected to HRCA for 45 min in PCR thermocycler at 30 °C.

### 2.7 Colorimetric Detection

To initiate the colorimetric detection of the HRCA product, 6 μL of a substrate solution was mixed with 6 μL of ABTS and 6 μL of H_2_O_2_. The presence of G-quadruplex structure after HRCA-amplification in the presence of hemin catalyzed the oxidation of ABTS by H_2_O_2_, resulting in the development of a green color. This colorimetric change was visually detectable and monitored by capturing digital images of the reaction mixtures at 30 s intervals. The captured images were processed using a Python-based image analysis pipeline to extract RGB values, which were subsequently converted to grayscale intensity values for quantitative evaluation of color development. This approach enabled real-time monitoring of the HRCA amplification and provided a simple, equipment-free method for signal readout.

### 2.8 Blood Sample Analysis

To evaluate the applicability of the developed HRCA-based detection system in a clinically relevant matrix, blood samples were then spiked with CK-MB to reach the final concentrations of 0.5 to 100 ng/mL. For selective target capture, each sample was incubated with MNP-apt under optimized binding conditions. Following incubation, magnetic separation and washing steps were performed to remove unbound components and reduce background signals. The HRCA reaction was then performed on the target-bound MNP-aptamer complexes under isothermal conditions for 45 min to amplify the detection signal. This procedure enabled specific and sensitive detection of CK-MB in a complex biological matrix, demonstrating the potential clinical utility of the assay.

### 2.9 Nanodrop Characterization

To verify aptamer binding, 20 μL MNPs were incubated with 5 μL aptamer. After incubation, 2 μL of the MNPs-aptamer mixture was analyzed via Nanodrop before and after removing supernatant. A decrease in DNA concentration confirmed successful binding.

### 2.10 TapeStation Electrophoresis Analysis

The amplification products of the HRCA reaction were analyzed using an Agilent TapeStation 4200 system equipped with High Sensitivity Genomic DNA ScreenTape assays. Samples were prepared according to the manufacturer’s instructions. Briefly, 1 μL of each HRCA product or control was mixed with 10 μL of High Sensitivity Genomic DNA sample buffer (1:10 ratio) and vortexed before analysis. For analysis of ligated padlock probe products, D1000 ScreenTape assays were used. In this case, 1 μL of the sample was mixed with 3 μL of D1000 sample buffer (1:3 ratio) according to the recommended protocol. The prepared mixtures were loaded into the ScreenTape wells along with the appropriate DNA ladder, and electrophoretic separation was performed under automated conditions. Data acquisition and analysis were carried out using Agilent TapeStation Analysis Software. Negative control samples prepared without CK-MB were included to evaluate background amplification.

### 2.11 Statistical Analysis

All statistical analyses were performed using a two-tailed Student’s *t*-test (two-sample, unequal variance). This approach was applied to compare differences between CK-MB samples and control or between individual experimental groups, without assuming equal variances. No assumption was made regarding the direction of the effect, and therefore, a two-tailed test was applied. Data are presented as mean ± standard deviation (SD) from three independent replicates (*n* = 3). Statistical significance was defined as *p* < 0.05 unless otherwise specified.

## 3 Results and Discussions

### 3.1 Theoretical Confirmation of the Sensing Mechanism

To computationally validate the secondary structure of the engineered aptamer, the Nucleic Acid Package (NUPACK) software suite was used to simulate folding behavior at an aptamer concentration of 1 μM. As shown in Figure 1B, in the absence of target the aptamer–splint complex remains in a closed conformation, stabilizing the secondary structure and suppressing nonspecific activation. Moreover, the addition of a poly-A (A_8_) spacer prevents unwanted hybridization and reduces steric hindrance. This modification improves the stability of the aptamer and allows efficient conformational switching upon target binding.^27^ To ensure specificity, the splint sequence was analyzed using BLAST to verify that it does not match any human genomic sequences, thereby preventing potential cross-amplification with host DNA. The BLAST results showed 100% identity between the sequence and the genomes from other species (Figure S1A). Table S1 summarizes the sequences of the nucleic acid components designed for the HRCA-based assay, including a CK-MB-specific aptamer selected for its high affinity and specificity toward the target, as reported in previous studies.^28^ This aptamer was engineered to incorporate locking sequences, a spacer, and a splint, as shown in Figure 1B. Moreover, the sequences of the padlock probe and primer are also included in Table S1. Next, NUPACK simulations were performed to investigate the interactions and thermodynamic properties of the engineered aptamer with the circular padlock probe and primer. Figure 1C shows the secondary structure of the padlock probe hybridized with the splint region of the engineered aptamer and primer. The mixture of padlock probe, splint, and primer exhibited a predicted minimum free energy of 43.89 kcal/mol, indicating strong thermodynamic stability. Figure 1D displays the strand displacement and the formation of G-quadruplex after RCA. The padlock probe contains sequences in it that are complementary to G-quadruplex (C), and after RCA, the actual G-quadruplex sequence will be created (C*). The base-pairing probability plot further confirmed high stability in stem-loop regions, with pairing probabilities exceeding 0.9 in critical binding domains. Complex formation analysis further demonstrated robust and specific interactions among the splint (strand 1), padlock probe (strand 2), and primer (strand 3). Equilibrium concentration simulations showed dominant formation of the complete three-strand HRCA initiation complex, with equilibrium levels exceeding 300 nM. In contrast, side complexes involving only individual strands or partial assemblies were detected at significantly lower concentrations, confirming minimal nonspecific interactions (Figure S1B). These results collectively validate the structural integrity of the padlock probe, splint, and primer and confirm their thermodynamic stability for initiating HRCA with high specificity and efficiency. Finally, the design of these components ensures efficient target recognition, probe circularization, and robust amplification for sensitive CK-MB detection.

### 3.2 Validating HRCA Amplicon Formation Using Gel Electrophoresis

After computational simulations predicted the expected HRCA product, amplification was experimentally verified using gel electrophoresis.

For successful circularization, the padlock probe was first hybridized with a complementary splint oligonucleotide at its 5′ and 3′ termini, enabling ligation to form circular DNA (Figure 2A). The annealing of the padlock probe occurs due to its phosphorylated 5′ end. After ligation, the circular padlock probe was treated with exonuclease III, which selectively digests single and double-stranded linear DNA to remove nonspecific interaction in the assay. By eliminating these linear DNA species, exonuclease treatment ensures that only fully circularized padlock probes serve as templates for amplification, improving assay specificity^29^ (Figure 2B). To evaluate the performance of splint-assisted ligation, reactions were performed at 25 °C using T4 DNA ligase. Figure 2C shows the TapeStation gel electrophoresis results for ligation reactions performed in the presence of 100 nM splint and a negative control without splint. A DNA ladder was used for size estimation. In the absence of the splint, a single sharp band at approximately 50 bp is observed, corresponding to the linear padlock probe. In contrast, the sample containing 100 nM splint exhibits a distinct band at approximately 300 bp with slower migration, along with higher-molecular-weight bands, indicating successful ligation and formation of circularized DNA products. The additional bands observed at approximately 90 bp, 150 bp, and 200 bp are attributed to partially ligated or intermediate products arising from incomplete hybridization or ligation.

**Figure 2:**
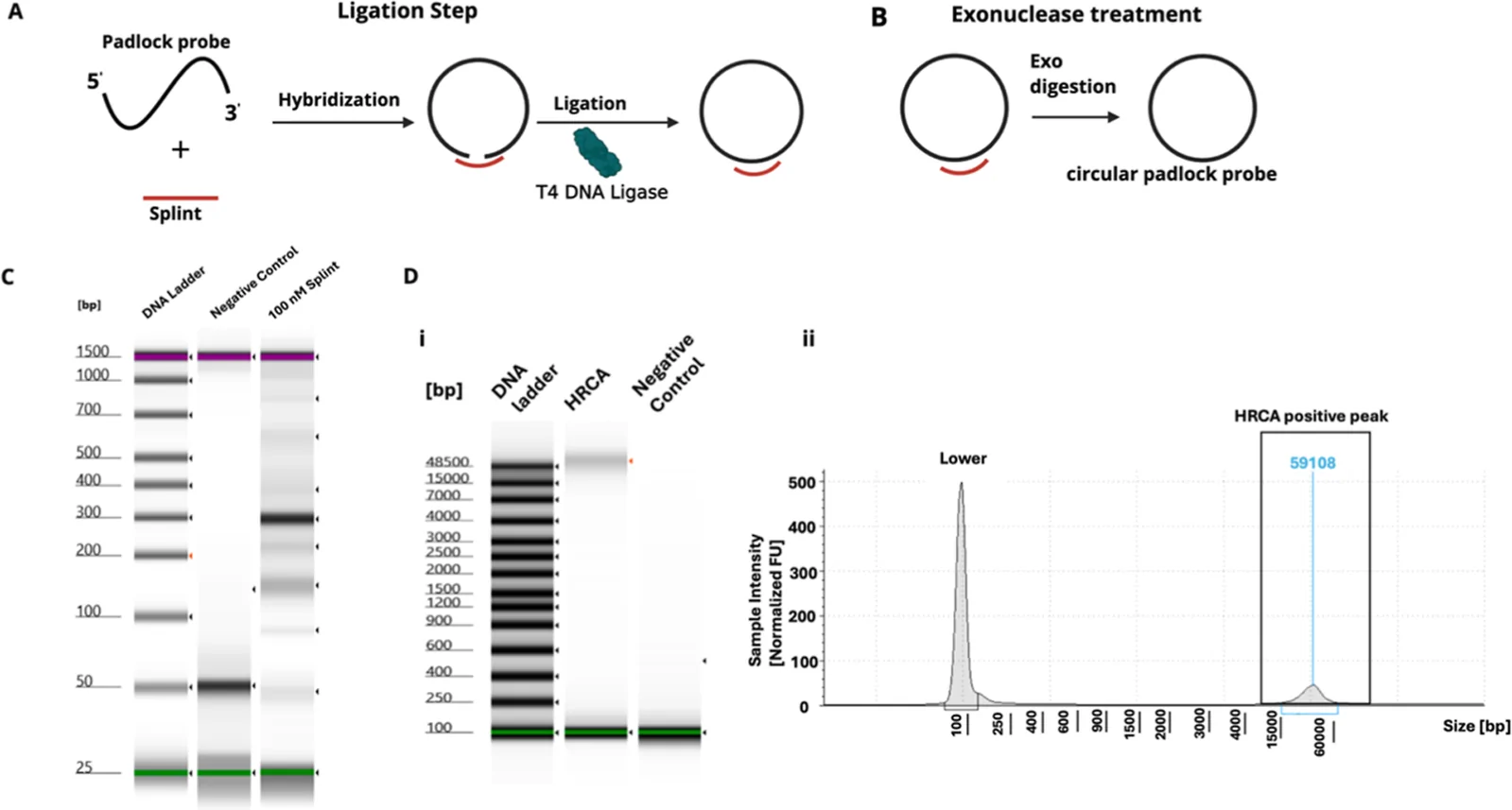
Verification of padlock probe circularization and HRCA amplification. (A) Schematic illustration of splint-assisted ligation of the padlock probe using T4 DNA ligase at 25 °C. Hybridization of the splint aligns the 5′ and 3′ termini of the probe to facilitate circularization. (B) Exonuclease digestion assay showing selective removal of linear padlock while preserving intact circular products. (C) TapeStation electrophoresis of splint-assisted padlock probe ligation: DNA ladder; padlock probe without splint (negative control); and splint-padlock mixture at 100 nM. The band at approximately 300 bp confirms circularization, with increased ligation efficiency observed in the presence of the splint. (D) (i) TapeStation electrophoresis confirming HRCA products. DNA ladder, positive HRCA reaction, showing high-molecular-weight amplicons (59 KB), and negative control with no detectable amplification. (ii) Electropherogram of the HRCA-positive sample (B1), revealing a dominant amplification peak at 59,108 bp.

By validating the splint concentration required for efficient ligation, we ensured that the system produces robust and specific HRCA products upon CK-MB binding, establishing a strong foundation for downstream signal generation. To further validate the efficiency and specificity of the optimized HRCA system, gel electrophoresis was performed to confirm successful amplification. As shown in Figure 2D­(i), the positive HRCA sample displayed a distinct high-molecular-weight band, and the electropherogram (TapeStation Analysis software from Agilent) revealed a major peak at 59,108 bp (Figure 2D­(ii)). This broad distribution of large DNA fragments confirms that the HRCA reaction successfully generated hyper-branched DNA structures in the presence of 100 ng/mL CK-MB. In contrast, the DNA ladder provided a size reference, while the negative control, prepared without CK-MB, showed only a baseline signal with no amplification peaks. The absence of amplification in the negative control, together with the sharp high-molecular-weight peak in the positive sample, provides strong evidence that HRCA was selectively initiated by CK-MB binding and generated large amounts of DNA. Collectively, these electropherograms confirm that the locked-aptamer HRCA platform is both robust and specific, yielding well-defined hyperbranched DNA amplicons suitable for downstream colorimetric readout and quantitative detection (Figure 2D). To verify the target-induced conformational switching of the locked aptamer, HRCA experiments were performed in both the absence and presence of CK-MB. This experiment was designed to confirm that the aptamer undergoes structural opening and initiates HRCA even at low concentrations of CK-MB. As shown in Figure S2A, in the control sample without CK-MB, no amplification bands were observed in TapeStation analysis, indicating that the aptamer remained in its locked conformation and that the internal splint sequence was not accessible for amplification. Upon the addition of 5 ng/mL CK-MB, a high-molecular-weight band (∼15,000 bp) was detected, confirming that target binding induced structural opening of the aptamer, thereby exposing the splint sequence and initiating HRCA.

To confirm the successful conjugation of aptamers to MNPs, a quantitative binding assay was performed, and the absorbance was measured using UV–vis spectrophotometry. The aptamer concentration was measured before binding and after incubation with MNPs (Figure S2B). Initial aptamer concentration in the stock solution was approximately 260 ng/μL. Upon incubation with the MNPs and subsequent magnetic separation, the unbound aptamer in the supernatant decreased to 75 ng/μL, while the bound aptamer was estimated to be 185 ng/μL, which demonstrates effective immobilization of the aptamer onto the MNPs surface. This binding is mediated through streptavidin–biotin affinity. These findings confirm that the magnetic nanoparticles were successfully functionalized and can serve as reliable carriers for aptamer–target complexes.

To evaluate the effect of the HRCA primer on amplification efficiency, HRCA reactions were performed in the presence and absence of the primer (*n* = 3), and UV–vis absorbance was measured at 420 nm. As shown in Figure S2C, reactions containing the HRCA primer exhibited significantly higher absorbance compared to those without the primer, indicating enhanced amplification. This result confirms that the HRCA primer promotes secondary strand synthesis and hyperbranched amplification, leading to an increased number of G-quadruplex-forming sequences and improved signal generation.

### 3.3 Determination of Assay Sensitivity

Following confirmation of successful HRCA amplification by gel electrophoresis, the peroxidase-like activity of the hemin- G-quadruplex generated during amplification was evaluated. To assess overall assay performance, aqueous samples were spiked with varying concentrations of CK-MB, followed by initiation of the colorimetric reaction through the addition of ABTS and H_2_O_2_. ABTS is a widely used peroxidase substrate due to its high-water solubility and its green coloration upon oxidation.^30^ Oxidation of ABTS was catalyzed by the G-quadruplex in the presence of hemin. Hemin is a catalytic cofactor, mimicking peroxidase activity and enabling the complex to efficiently catalyze electron transfer reactions (Figure 3A­(i)). Compared to traditional enzymes such as horseradish peroxidase (HRP), this catalytic G-quadruplex complex offers superior chemical stability, thermal resistance, and cost-effectiveness, making them especially advantageous for colorimetric reaction and long-term storage. To enhance and accelerate the colorimetric signal, H_2_O_2_ was added 160 s after ABTS. H_2_O_2_ acts as the final electron acceptor in the catalytic cycle, boosting the oxidation of ABTS and intensifying the resulting color change. A colorimetric reader (Figure 3A­(ii)) was developed as a compact, low-cost imaging system for quantitative signal analysis. The assay was performed in a cylindrical, flat-bottom sample tube inserted into a 3D-printed holder made of opaque acrylic to minimize ambient light interference. A magnetic collar embedded with an N52 magnet was positioned around the holder to facilitate magnetic separation of HRCA-amplified magnetic nanoparticles, pulling them to the bottom of the tube and ensuring a clear optical path. The holder was mounted on a sample stage equipped with an LED backlight panel positioned beneath the tube to provide uniform and stable illumination. The illumination system was powered by a simple electronic circuit consisting of a switch, a rechargeable battery, and a variable resistor to adjust light intensity. A manual-focus webcam was mounted above the sample in a 3D-printed lid, aligned directly with the tube to capture transmitted light images. The lid was magnetically coupled to the base to ensure proper alignment and effective shielding from external light. Images were acquired at user-defined time intervals (typically every 30 s) and processed in real time using a custom Python-based program with a graphical user interface for RGB signal extraction and analysis.^22^

**Figure 3:**
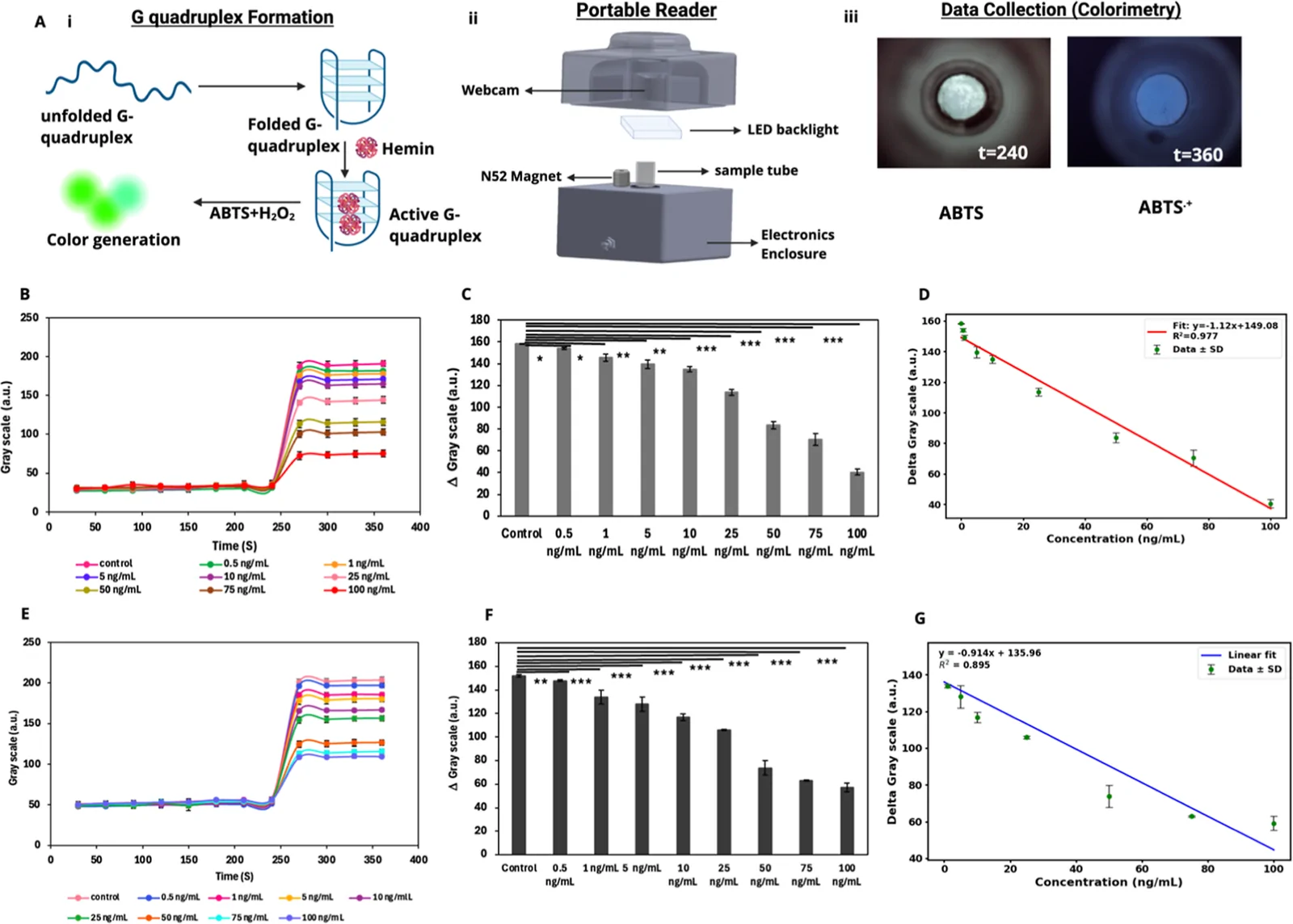
Development and optimization of the HRCA-based colorimetric detection platform for CK-MB. (A)­(i) Schematic of the G-quadruplex formation and colorimetric reaction. (ii) Schematic of the portable colorimetric reader. (iii) Color changes of the assay before and after magnetic separation in the presence of ABTS/H_2_O_2_. (B) Time-resolved grayscale intensity profiles for CK-MB detection at concentrations ranging from 0.5 ng/mL to 100 ng/mL in aqueous solution, compared with the control sample. (C) Δ grayscale values for different CK-MB concentrations in aqueous solution, showing a decrease with increasing CK-MB concentration. Statistical significance was assessed using a two-tailed Student’s *t*-test (*p* < 0.05, *p* < 0.01, *p* < 0.001). (D) Calibration curve of CK-MB in aqueous solution within the range of 0.5 to 100 ng/mL, showing Δ grayscale values plotted against CK-MB concentrations. (E) Time-resolved grayscale intensity for CK-MB detection at concentrations ranging from 0.5 ng/mL to 100 ng/mL in blood-spiked samples, compared with the control sample. (F) Δ grayscale values for different CK-MB concentrations in blood-spiked samples, showing a decrease with increasing CK-MB concentration. Statistical significance was assessed using a two-tailed Student’s *t*-test (*p* < 0.01, *p* < 0.001). (G) Grayscale-based calibration in whole blood samples spiked with CK-MB (0.5–100 ng/mL). (Each data point indicates the mean of three independent replicates (*n* = 3), with error bars representing the SD).

In the presence of CK-MB, target-dependent generation of G-quadruplex structures catalyzed the peroxidase reaction, producing a visible green color that intensified over time (Figure 3A­(iii)), and its intensity was recorded at every 30 s using a colorimetric reader machine. RGB intensity values were measured by the colorimetric reader machine every 30 s before and after H_2_O_2_ addition, with the green channel showing a proportional increase corresponding to higher CK-MB concentrations. The program extracts RGB intensity values from each image, displaying both the live sample view and a dynamic plot of colorimetric changes over time. For simplified and more robust data analysis, the RGB values were also converted to grayscale using the standard luminance formula^31^ (eq 1).

1 Grayscale=0.2989R+0.5870G+0.1140B

Using grayscale values instead of individual RGB channels reduced dimensionality, minimized variability from lighting and color artifacts, and improved consistency across imaging sessions. Greyscale also showed a stronger correlation with concentration-dependent absorbance changes, making it more suitable for quantitative analysis.^32^ Therefore, grayscale intensity was used as the primary metric for generating calibration curves, calculating the LOD, and evaluating overall assay performance. Figure 3B shows the grayscale intensity profiles of the HRCA-based colorimetric assay in aqueous solution with varying concentrations of CK-MB, including 0.5 ng/mL, 1 ng/mL, 5 ng/mL, 10 ng/mL, 25 ng/mL, 50 ng/mL, 75 ng/mL, and 100 ng/mL, compared to a control sample without CK-MB. Grayscale values were extracted from RGB images captured at 30 s intervals, offering a quantitative readout of the colorimetric signal over time. The experiment was repeated three times, and the error bars represent the SD of the measurements. As shown, all CK-MB-spiked samples displayed a noticeable increase in grayscale intensity beginning around 240 s after the addition of the ABTS/H_2_O_2_ substrate. This increase corresponds to the visible development of a green color, catalyzed by the G-quadruplex formed during HRCA amplification. Importantly, grayscale intensity showed an inverse correlation with CK-MB concentration, and higher CK-MB levels produced a darker green color, which yielded lower grayscale values. Among the samples, the 100 ng/mL condition showed the smallest increase in grayscale intensity relative to the control, consistent with the production of a darker green color and correspondingly lower grayscale values. These findings confirm the assay’s high specificity and dose-dependent response, validating the HRCA platform as a rapid and sensitive method for CK-MB detection. The Δ grayscale value for each CK-MB concentration in aqueous solution was calculated by subtracting the average grayscale value after magnetic separation from the average grayscale value before magnet addition. As shown in Figure 3C, the Δ grayscale value decreased as the CK-MB concentration increased from 0.5 to 100 ng/mL. The control sample exhibited the largest grayscale difference, indicating no color generation, whereas higher CK-MB concentrations produced a darker green color and smaller grayscale differences. The error bars represent SD from three replicate measurements, and Student’s *t*-test was performed to compare each concentration with the control group. The bar graph shows that even low concentrations of CK-MB showed a statistically significant decrease compared with the control (*p* < 0.05­(*)), and the difference becomes increasingly significant at higher concentrations (*p* < 0.01­(**) and *p* < 0.001­(***)). These results confirm that the grayscale intensity changes are statistically significant and concentration-dependent, demonstrating the assay’s sensitivity and quantitative reliability. Furthermore, the calibration plot of CK-MB concentrations ranging from 0.5 to 100 ng/mL in aqueous solution, based on the Δ grayscale value, exhibited a linear relationship with an *R* ^2^ of 0.977 and regression equation of *y* = −1.12*x* + 149.08, as shown in Figure 3D. To evaluate sensitivity of the developed HRCA-based biosensing system for CK-MB detection, the LOD and LOQ were calculated based on the grayscale intensity values obtained from the colorimetric reader after magnetic separation in aqueous samples. The LOD was determined using the widely accepted 3σ criterion, as defined in eq 2,^33^ while the LOQ was calculated using the 10σ criterion, as shown in eq 3.^34^

2 LOD=3×σblank/(slopeofthecalibrationcurve)

3 LOQ=10×σblank/(slopeofthecalibrationcurve)

Here, σ_blank represents the standard deviation of the grayscale signal from the blank (no CK-MB) and was equal to 0.1. The slope was obtained from the linear calibration curve fitted using a regression model (*R* ^2^ = 0.977). Based on this analysis, the LOD and LOQ for CK-MB detection in aqueous samples were calculated to be 0.268 ng/mL and 0.89 ng/mL, respectively. To enhance the efficiency of the HRCA-based colorimetric assay for CK-MB detection, a two-step optimization process was performed, focusing on reaction time and padlock probe concentration. In the first step, HRCA was carried out using a fixed padlock probe concentration of 10 nM over multiple time intervals (45 min, 2, 3, and 4 h). Following each reaction, the colorimetric output was evaluated by adding ABTS and H_2_O_2_.Figure S3A). However, for clinical translation, minimizing the total assay time is critical. To address this, a second optimization step was conducted to assess whether increasing the padlock probe concentration could compensate for the shortened amplification duration. The assay was performed at 45 min using varying circular padlock probe concentrations, including 0.1 nM, 10 nM, 100 nM, and 500 nM with 25 ng/mL CK-MB. The results showed a clear increase in signal intensity with increasing padlock concentration. Notably, the assay performed with 500 nM padlock at 45 min produced a grayscale output nearly equivalent to that obtained with 10 nM probe at 4 h, with grayscale values of 70 (Figure S3B). To cross-check the amplification efficiency of the HRCA-based colorimetric assay, UV–vis absorbance measurements were performed at 475 nm for the same assay after a 45 min HRCA reaction (Figure S3C). The absorbance values corresponding to these concentrations were approximately 0.45, 0.53, 0.68, and 0.77, respectively. A clear concentration-dependent increase in absorbance was observed, indicating enhanced production of G-quadruplex structures capable of catalyzing the oxidation of ABTS in the presence of H_2_O_2_, thereby generating a stronger green color signal. Notably, the highest padlock probe concentration of 500 nM produced the greatest absorbance value of 0.77. Student’s *t*-test was performed to compare the UV–vis absorbance at different padlock probe concentrations, using 0.1 nM as the reference. Statistically significant differences were observed for all concentrations (*P* < 0.05), with increasing significance at higher concentrations. Specifically, *P* < 0.05 for 10 nM, *P* < 0.01 for 100 nM, and *P* < 0.001 for 500 nM. This finding demonstrates a significant improvement in signal intensity and assay speed, highlighting 500 nM as the optimal concentration for the rapid and sensitive detection of CK-MB. All optimization experiments were conducted in triplicate, and standard deviations were calculated and included in the graph.

To visually validate the performance of the HRCA assay in blood samples, blood was spiked with different concentrations of CK-MB (0.5–100 ng/mL), followed by a 45 min HRCA assay. The experiment was performed in triplicate, and error bars represent the standard deviation. As shown in Figure 3E, a colorimetric reaction was performed by the addition of ABTS and H_2_O_2_, followed by magnetic separation, which initiated the creation of green color and subsequent increase in grayscale intensity. As with the aqueous solutions, there is an inverse correlation between the increasing concentration of CK-MB in blood samples and the grayscale value. Figure 3F shows a decrease in Δ grayscale values as the CK-MB concentration increased from 0.5 to 100 ng/mL. Error bars represent the SD, and each experiment was performed in triplicate (*n* = 3). Statistical significance was assessed using a two-tailed Student’s *t*-test, demonstrating significant differences between CK-MB concentrations in blood-spiked samples. Subsequently, the calibration plot for CK-MB detection in blood samples is presented in Figure 3G. The sensitivity of the assay was evaluated by calculating the LOD according to eq 2 and the LOD according to eq 3. Here, σ_blank was 0.1, and the calibration curve exhibited a *R* ^2^ of 0.895. The LOD and LOQ were calculated to be 0.328 ng/mL and 1.09 ng/mL, respectively, in blood-spiked samples, demonstrating high analytical sensitivity and enabling detection below the clinical cutoff for myocardial injury. To assess the practicality of this assay, the colorimetric response of 22 blood samples spiked with CK-MB was investigated, and the obtained grayscale values were fitted to the calibration curve for CK-MB detection in blood. Based on the linear calibration equation, the CK-MB concentrations in the blood samples were determined. As shown in Table 1, the recoveries ranged from 100% to 114%. The Relative Standard Deviations (RSDs), calculated according to eq 4,^35^ where δ represents SD of the sample and x̅ is the mean, were low (3.93%, *n* = 3), indicating good precision. These findings validate the potential of the assay for real-world applications, particularly in acute coronary syndrome, where biomarker levels typically exceed this LOD. The successful demonstration in blood further supports the assay’s robustness and its potential to be extended to other cardiac biomarkers using a similar platform. To evaluate the specificity of this experiment, the HRCA was performed in the presence of myoglobin, cTnI, and cTnT. As shown in Figure S3D, the Δ grayscale values remained very low for other biomarkers (around 20). However, for CK-MB, the Δ grayscale value was significantly different (approximately 81), with *P* < 0.001. These results demonstrate the high specificity of the assay for CK-MB detection, confirming its practical applicability and indicating no interference from other biomarkers.

4 $$RSD=\left(\frac{\delta }{\mathrm{\chi ̅}}\right)\times 100$$

**Table 1:** Analytical Results for CK-MB Monitoring in 22 Blood-Spiked Samples (*n* = 3)^a^

sample (blood)	amount added (ng/mL)	amount found (ng/mL)	recovery (%)	RSD (%) (*n* = 3)
1	0.5	0.57	114	0.75
2	1	1.1	110	0.94
3	5	5.5	110	3.02
4	10	10.5	105	2.52
5	15	15	100	0.96
6	20	22	110	3.71
7	25	28	112	2.63
8	30	33	110	0.54
9	35	35	100	3.93
10	40	42	105	1.65
11	45	46	102	3.26
12	50	52	104	3.54
13	55	58	105	2.45
14	60	62	103	3.12
15	65	67	103	2.57
16	70	71	101	1.02
17	75	77	103	2.34
18	80	84	105	3.54
19	85	87	102	3.76
20	90	92	102	2.43
21	95	96	101	4.12
22	100	100	100	3.66

a Each sample corresponds to a different CK-MB concentration.

## 4 Conclusion

In summary, a rapid and integrated workflow for CK-MB detection was established and validated, combining splint-assisted padlock ligation, HRCA, and a portable colorimetric reader. Circularization efficiency was improved by optimizing ligation at 25 °C with 100 nM splint concentrations. The colorimetric reaction was performed using ABTS/H_2_O_2_ oxidation, and the RGB values of each color were measured using a colorimetric reader. The assay demonstrated excellent sensitivity, with LOD values of 0.268 and 0.328 ng/mL and LOQ values of 0.89 and 1.09 ng/mL in aqueous and blood-spiked samples, respectively. The assay exhibited stable performance when key components were stored under appropriate conditions (−20 and 4 °C) for up to one month. Although aptamer-based biosensors have been reported for the detection of cardiac biomarkers such as CK-MB, cTnI, cTnT, and myoglobin,^36^ many existing approaches rely on multistep amplification strategies or complex nanomaterial-assisted signal transduction. Recent studies have demonstrated the integration of aptamers with RCA for the detection of cardiac biomarkers, including CK-MB.^37^ Moreover, aptamer-RCA platforms have been widely explored for cTnI detection, achieving ultralow detection limits in the pg/mL range with fluorescence-based approaches. However, these systems often involve complicated fluorescence instrumentation and multicomponent nanomaterial systems, which may limit their practical applicability.^38^ In contrast, the present work introduces a locked aptamer-based HRCA strategy, enabling target-induced activation and signal amplification in a simplified system. Although the detection limit is in the ng/mL range, this sensitivity is well aligned with the clinically relevant concentration range of CK-MB, making the assay suitable for practical diagnostic applications. Moreover, the use of HRCA provides enhanced signal amplification efficiency, while the locked aptamer design improves specificity, offering a robust and straightforward platform for cardiac biomarker detection. When compared with commercial platforms (Siemens, Roche, Abbott), which generally employ electrochemiluminescence, electrochemical detection, fluorescence immunoassays, and colorimetric formats (Table 2), the HRCA-based assay developed in this study demonstrated LOD of 0.328 while requiring minimal instrumentation. A visible readout was produced in less than 2 min, and the total assay time was 45 min through the integration of aptamer-mediated recognition, isothermal HRCA amplification, and G-quadruplex-induced color development. This platform represents a versatile and modular approach that can be adapted to a wide range of biomarkers. Its nucleic acid programmability, portability, compatibility with whole blood, and minimal infrastructure requirements highlight its strong potential for POC diagnostics, particularly in multiplexed biosensing systems. The design enables extension to multiplexed detection of cardiac biomarkers such as cTnI, cTnT, and myoglobin by incorporating target-specific aptamer sequences. Multiplexing can be achieved through spatial separation of detection zones or by employing distinct chromogenic substrate systems to generate differentiable colorimetric signals, demonstrating the scalability and flexibility of the assay. Although the assay was successfully validated using blood-spiked samples, further validation with clinical patient samples is required to establish its clinical applicability. Future studies will focus on evaluating performance in real clinical settings, including samples from patients with AMI and healthy controls. Such validation will enable a comprehensive assessment of diagnostic sensitivity, specificity, and robustness in complex physiological environments, which is essential for translating the proposed HRCA-based colorimetric platform into practical clinical use.

**Table 2:** Comparison of Various Commercial CK-MB Detection Systems with the Present Study

name of the system	readout	dynamic range (ng/mL)	limit of detection (ng/mL)	additional instrument requirement	reference
Siemens (Acute Care CK-MB)	fluorescence immunoassay	0.3–150	0.3	yes	^39^
Elecsys CK-MB STAT	electrochemiluminescence immunoassay (ECLIA)	0.3–300	0.3	yes	^40^
AFIAS Boditech	fluorescence	3–100	1.17	yes	^10^
i-STAT CK-MB	electrochemical	0–150	N/A	yes	^41^
Anbio CK-MB rapid test	fluorescence immunoassay	2–100	N/A	yes	^42^
CK-MB Combo FIA	fluorescence	1–200	N/A	yes	^43^
Human CKMB ELISA Kit	colorimetric	0.328–80	0.3	yes	^44^
Finecare CK-MB Rapid test	fluorescence immunoassay	0.3–100	N/A	yes	^45^
this study	colorimetric	0.5–100	0.328	no	-

## Supplementary Material



## Notes

Similarity validation and equilibrium concentration simulations of the splint, padlock probe, and primer; sequences of the designed oligonucleotides; TapeStation analysis of HRCA products; quantification of aptamer immobilization on magnetic nanoparticles by UV–vis spectroscopy; evaluation of primer effects on HRCA amplification efficiency; optimization of HRCA reaction conditions for enhanced colorimetric performance, including the effects of reaction time and padlock probe concentration on grayscale intensity; UV–vis absorbance spectra of HRCA products obtained at different padlock probe concentrations; and specificity studies (PDF)
