# Supplementary material for: A Hyperbranched Rolling Circle Amplification Assay Using Aptamer–Hemin/G-Quadruplex Recognition for the Detection of Creatine Kinase-MB
Source: ACS Meas Sci Au. 2026 Jul 1;6(4):972–82. doi: 10.1021/acsmeasuresciau.6c00016 (PMC13495452; doi:10.1021/acsmeasuresciau.6c00016)
Supplement: Supplementary file 1 [file tg6c00016_si_001.pdf]

A Hyperbranched Rolling Circle Amplification Assay Using Aptamer–Hemin/G-Quadruplex Recognition for the Detection of Creatine Kinase-MB

Ensiyeh Mirsadoughi [ab], **Sayantan Tripathy** [ab], **Fatemeh, Zare** [bc], **Gerard L Cote** [abc], **Samuel Mabbott**[ab]*

[a] Department of Biomedical Engineering, Texas A&M University, 101 Bizzell Street, College Station, TX 77843, USA

[b] Center for Remote Health Technologies & Systems, Texas A&M Engineering Experiment Station, 600 Discovery Drive, College Station, TX 77840-3006, USA

[c] Department of Electrical and Computer Engineering, Texas A&M University, College Station, TX, 77840, USA

* **Corresponding author:** smabbott@tamu.edu

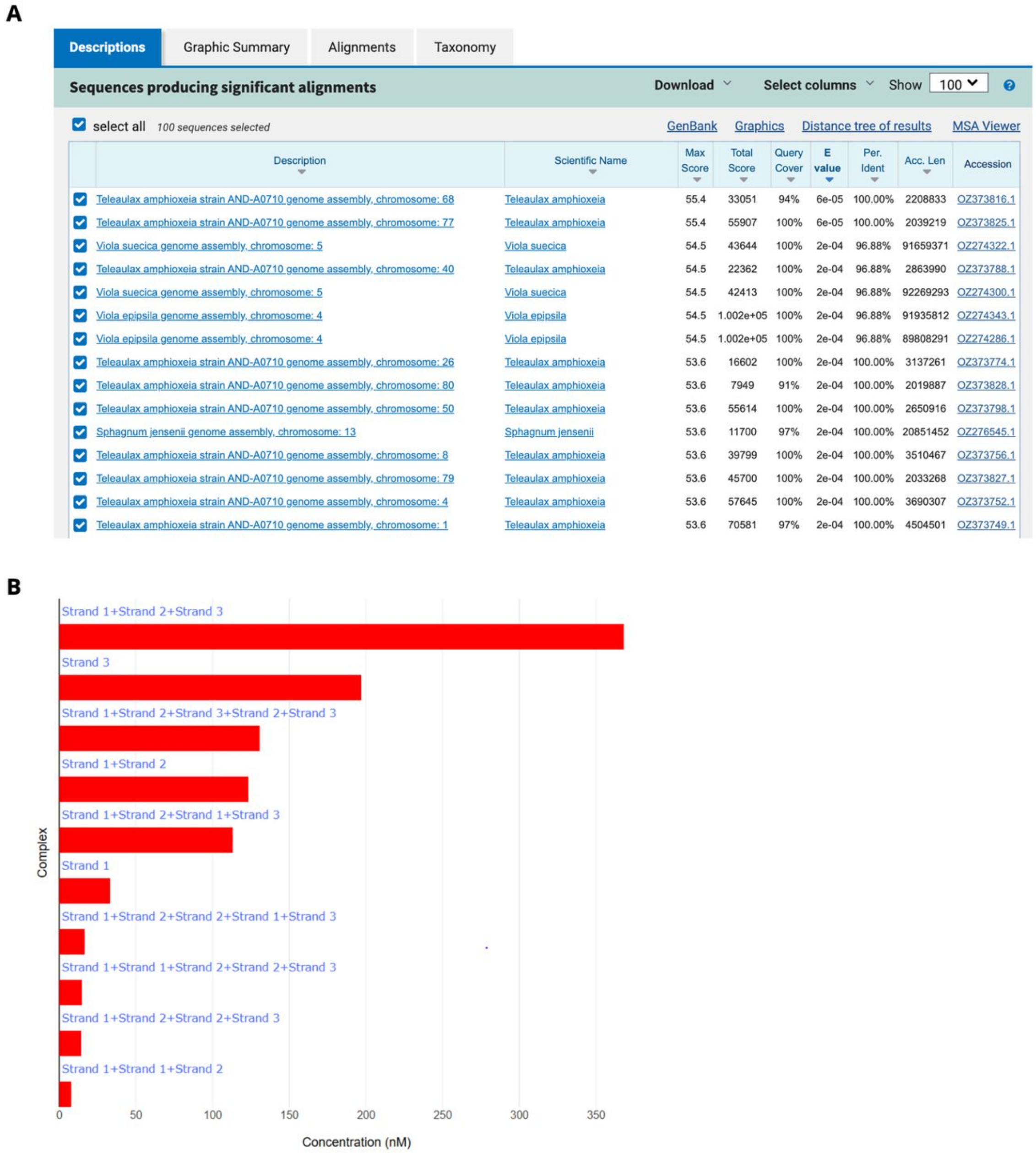

A

Descriptions | Graphic Summary | Alignments | Taxonomy

Sequences producing significant alignments — Download — Select columns — Show 100

select all 100 sequences selected — GenBank Graphics Distance tree of results MSA Viewer

Description	Scientific Name	Max Score	Total Score	Query Cover	E value	Per. Ident	Acc. Len	Accession
Teleaulax amphioxeia strain AND-A0710 genome assembly, chromosome: 68	Teleaulax amphioxeia	55.4	33051	94%	6e-05	100.00%	2208833	OZ373816.1
Teleaulax amphioxeia strain AND-A0710 genome assembly, chromosome: 77	Teleaulax amphioxeia	55.4	55907	100%	6e-05	100.00%	2039219	OZ373825.1
Viola suecica genome assembly, chromosome: 5	Viola suecica	54.5	43644	100%	2e-04	96.88%	91659371	OZ274322.1
Teleaulax amphioxeia strain AND-A0710 genome assembly, chromosome: 40	Teleaulax amphioxeia	54.5	22362	100%	2e-04	96.88%	2863990	OZ373788.1
Viola suecica genome assembly, chromosome: 5	Viola suecica	54.5	42413	100%	2e-04	96.88%	92269293	OZ274300.1
Viola epipsila genome assembly, chromosome: 4	Viola epipsila	54.5	1.002e+05	100%	2e-04	96.88%	91935812	OZ274343.1
Viola epipsila genome assembly, chromosome: 4	Viola epipsila	54.5	1.002e+05	100%	2e-04	96.88%	89808291	OZ274286.1
Teleaulax amphioxeia strain AND-A0710 genome assembly, chromosome: 26	Teleaulax amphioxeia	53.6	16602	100%	2e-04	100.00%	3137261	OZ373774.1
Teleaulax amphioxeia strain AND-A0710 genome assembly, chromosome: 80	Teleaulax amphioxeia	53.6	7949	91%	2e-04	100.00%	2019887	OZ373828.1
Teleaulax amphioxeia strain AND-A0710 genome assembly, chromosome: 50	Teleaulax amphioxeia	53.6	55614	100%	2e-04	100.00%	2650916	OZ373798.1
Sphagnum jensenii genome assembly, chromosome: 13	Sphagnum jensenii	53.6	11700	97%	2e-04	100.00%	20851452	OZ276545.1
Teleaulax amphioxeia strain AND-A0710 genome assembly, chromosome: 8	Teleaulax amphioxeia	53.6	39799	100%	2e-04	100.00%	3510467	OZ373756.1
Teleaulax amphioxeia strain AND-A0710 genome assembly, chromosome: 79	Teleaulax amphioxeia	53.6	45700	100%	2e-04	100.00%	2033268	OZ373827.1
Teleaulax amphioxeia strain AND-A0710 genome assembly, chromosome: 4	Teleaulax amphioxeia	53.6	57645	100%	2e-04	100.00%	3690307	OZ373752.1
Teleaulax amphioxeia strain AND-A0710 genome assembly, chromosome: 1	Teleaulax amphioxeia	53.6	70581	97%	2e-04	100.00%	4504501	OZ373749.1

Figure S1. Sequence similarity validation of splint and Equilibrium concentration simulations. (A) Validation of the splint sequence against the human genome showed no similarity, while 100% similarity was observed with sequences from other species. **(B)** Equilibrium concentration simulations of the splint (Strand 1), padlock probe (Strand 2), and primer (Strand 3), showing the formation of the complete three-strand HRCA complex, with equilibrium concentrations exceeding 300 nM.

Table S1: Sequences of designed oligonucleotides

Name	Sequence (5’⟶ 3’)	Modification/Description
Aptamer	5’TGGGCACACTTACAGTGGAAATTCGGC GTGGTTCTTCTTCGCTG3’	None
Locking sequence	TCAGCGAA	None
Spacer (Poly-A)	AAAAAAAA	Poly-A spacer
Splint	CCCTAAACCCTAAACCCTAAACCCTCAG CGAA	None
Padlock probe	5Phos/TTAGGGTTTAGGGAGCTCGGCTAG GC/TAGACCCTCCCTCCCTCCCTAGCT/GC TACTGACCCTAA/GGGTTTAGGGT	5′ phosphorylation required for ligation
HRCA Primer	CTAGCCGAGCT	None

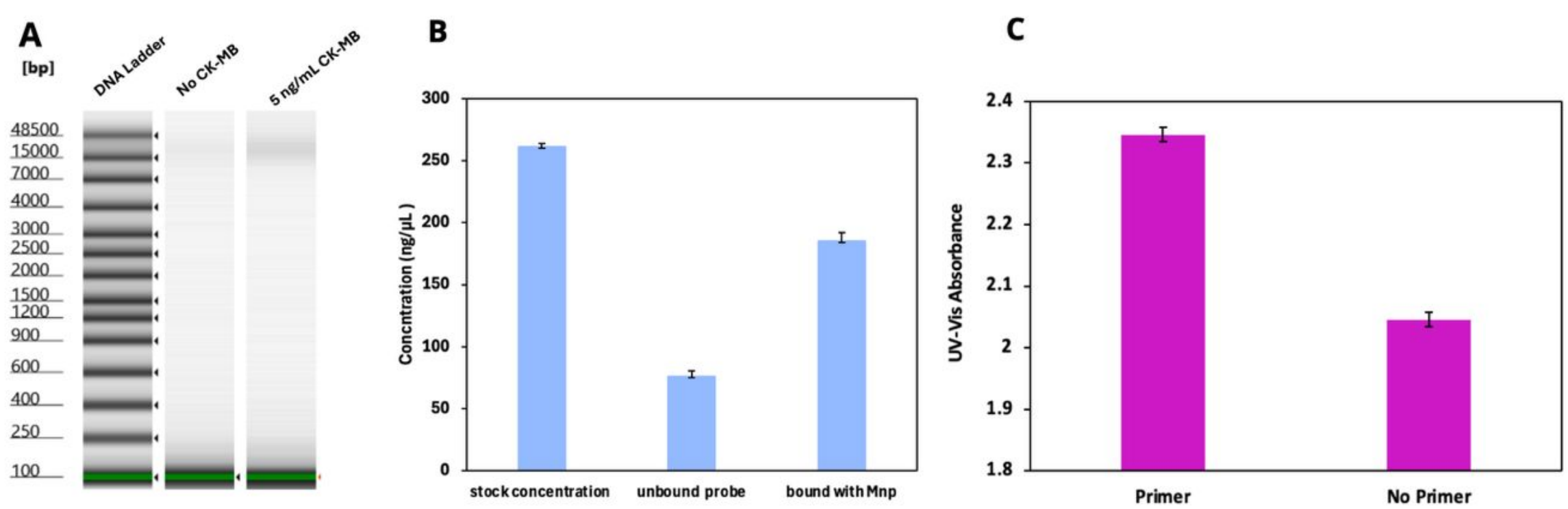

Figure S2. Validation of HRCA activation, aptamer immobilization, and primer effect.
(A) TapeStation gel electrophoresis analysis of HRCA activation via target-induced conformational switching of the locked aptamer. Lanes correspond to the DNA ladder, negative control without CK-MB, and 5 ng/mL CK-MB. No amplification is observed in the absence of CK-MB, while a high-molecular-weight band (~15000 bp) appears in its presence, confirming aptamer opening and HRCA initiation. (**B**) Quantification of aptamer binding to MNPs by UV-Vis absorbance. Aptamer concentrations were measured before binding, after incubation with MNPs to determine unbound aptamer levels, and after magnetic separation to assess bounded aptamers. The significant reduction in unbound aptamer indicates efficient immobilization on MNPs (n = 3, error bars = SD). **(C)** Effect of the HRCA primer on amplification efficiency, with higher UV–Vis absorbance at 420 nm observed in the presence of the primer, confirming enhanced amplification (n = 3, error bars represent SD).

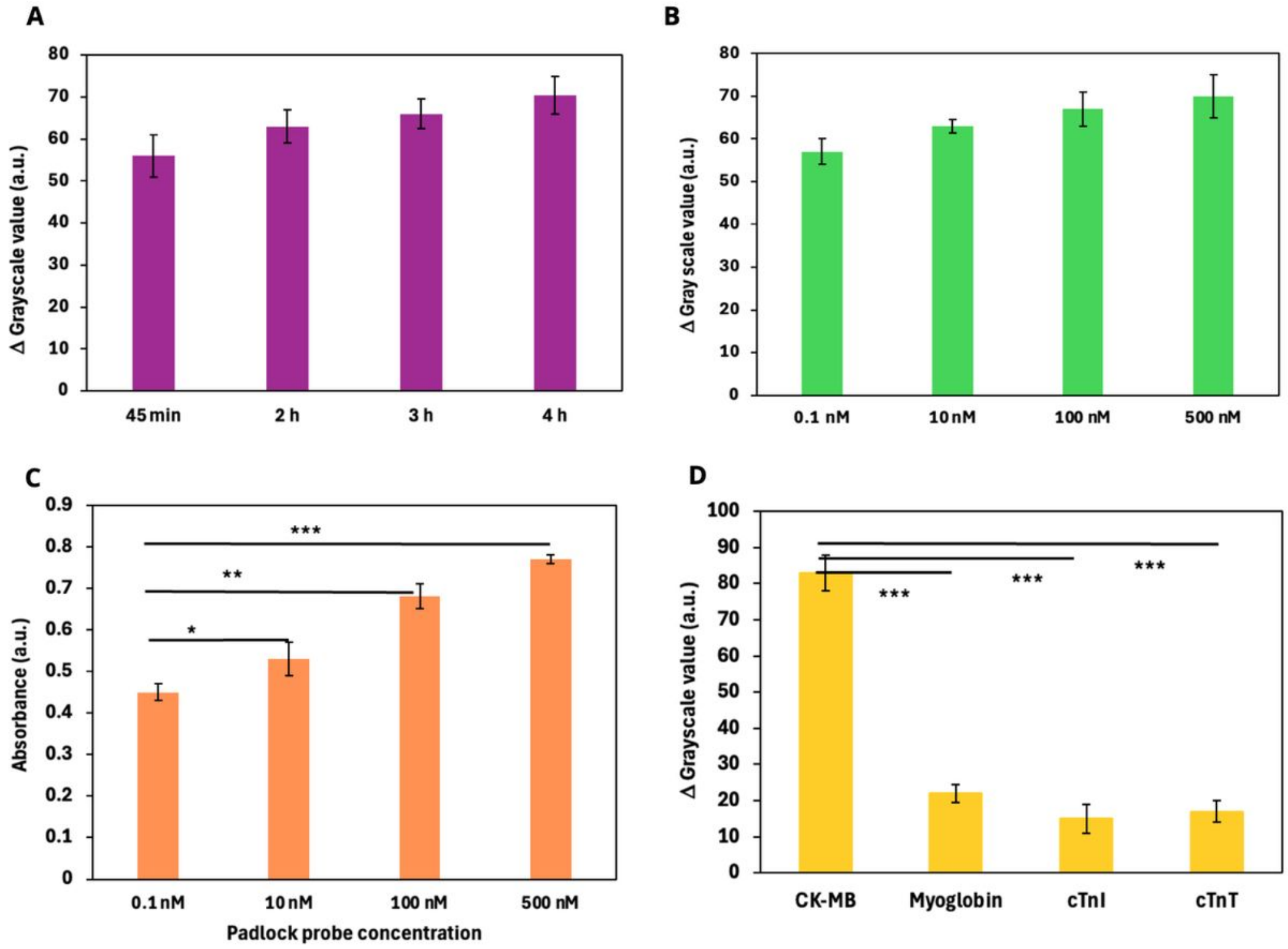

Figure S3. Optimization of HRCA reaction conditions for enhanced colorimetric signal. (A) Effect of reaction time on grayscale intensity at a fixed padlock probe concentration (10 nM). HRCA reactions were carried out for 45 min, 2 h, 3 h, and 4 h, followed by ABTS/H_2O_2 addition and measurement using the custom colorimetric reader. Error bars show standard deviation of three replicates. **(B)** Effect of padlock probe concentration on grayscale intensity at a fixed reaction time (45min). Reactions were performed using 0.1 nM, 10 nM, 100 nM, and 500 nM probe concentrations. Increasing padlock concentration significantly enhanced signal output, with 500 nM yielding nearly equivalent grayscale values to a 10 nM probe after 4 h amplification. Data represents the mean SD of three independent replicates. **(C)** UV-visible absorbance spectra of HRCA products after a 45 min reaction with varying padlock probe concentrations (0.1-500 nM), showing a clear concentration-dependent increase in signal intensity. Statistical significance was assessed using a two-tailed Student's t-test, with $p < 0.05$, $p < 0.01$, and $p < 0.001$ corresponding to padlock probe concentrations of 10, 100, and 500 nM, respectively. (**D**) Specificity evaluation across CK-MB, cTnI, cTnT, and myoglobin. Δ grayscale values were calculated after the colorimetric reaction, showing significant differences between CK-MB and the other biomarkers. Statistical significance was assessed using a two-tailed Student's t-test ($p < 0.001$ for myoglobin, cTnI, and cTnT). (All data were obtained from three replicates (n = 3), and error bars indicate the SD.)
